# Coutilization of glucose and glycerol enhances the production of aromatic compounds in an *Escherichia coli *strain lacking the phosphoenolpyruvate: carbohydrate phosphotransferase system

**DOI:** 10.1186/1475-2859-7-1

**Published:** 2008-01-22

**Authors:** Karla Martínez, Ramón de Anda, Georgina Hernández, Adelfo Escalante, Guillermo Gosset, Octavio T Ramírez, Francisco G Bolívar

**Affiliations:** 1 Departamento de Ingeniería Celular y Biocatálisis, Instituto de Biotecnología, Universidad Nacional Autónoma de México (UNAM), Av. Universidad 2001 CP 62210, Cuernavaca, Morelos, México; 2 Departamento de Medicina Molecular y Bioprocesos, Instituto de Biotecnología, Universidad Nacional Autónoma de México (UNAM), Av. Universidad 2001 CP 62210, Cuernavaca, Morelos, México

## Abstract

**Background:**

*Escherichia coli *strains lacking the phosphoenolpyruvate: carbohydrate phosphotransferase system (PTS) are capable of coutilizing glucose and other carbon sources due to the absence of catabolite repression by glucose. In these strains, the lack of this important regulatory and transport system allows the coexistence of glycolytic and gluconeogenic pathways. Strains lacking PTS have been constructed with the goal of canalizing part of the phosphoenolpyruvate (PEP) not consumed in glucose transport to the aromatic pathway. The deletion of the *ptsHIcrr *operon inactivates PTS causing poor growth on this sugar; nonetheless, fast growing mutants on glucose have been isolated (PB12 strain). However, there are no reported studies concerning the growth potential of a PTS^- ^strain in mixtures of different carbon sources to enhance the production of aromatics compounds.

**Results:**

PB12 strain is capable of coutilizing mixtures of glucose-arabinose, glucose-gluconate and glucose-glycerol. This capacity increases its specific growth rate (μ) given that this strain metabolizes more moles of carbon source per unit time. The presence of plasmids pRW300*aroG*^*fbr *^and pCL*tktA *reduces the μ of strain PB12 in all mixtures of carbon sources, but enhances the productivity and yield of aromatic compounds, especially in the glucose-glycerol mixture, as compared to glucose or glycerol cultures. No acetate was detected in the glycerol and the glucose-glycerol batch fermentations.

**Conclusion:**

Due to the lack of catabolite repression, PB12 strain carrying multicopy plasmids containing *tktA *and *aroG*^*fbr *^genes is capable of coutilizing glucose and other carbon sources; this capacity, reduces its μ but increases the production of aromatic compounds.

## Background

**Figure 1:**
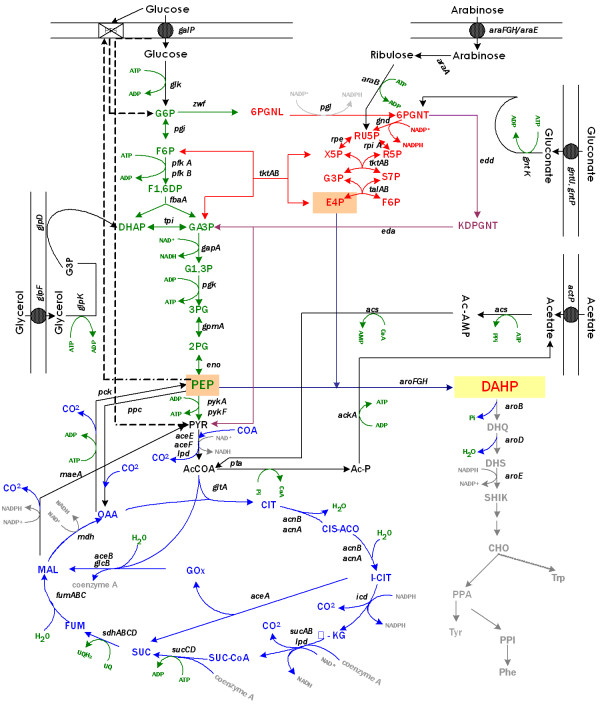
**Central metabolic and aromatic pathways**. The figure shows key metabolites, genes involved in their transformation, and genes coding for carbon transporters for those carbon sources coutilized by strain PB12. In this strain PTS is not functional. PB12 utilizes GalP permease and Glk for glucose transport and phosphorylation, respectively. Therefore, PEP that is not used for this purpose can be canalized for aromatic production [6,7]. Broken lines indicate the usual role and synthesis of some metabolites in the presence of PTS. Abbrevations are: glucose-6-phosphate (G6P), fructose-6-phosphate (F6P), fructose-1,6-phosphate (F1,6P), dihydroxy-acetone-phosphate (DHAP), glyceraldehyde-3-phosphate (GA3P), glyceraldehyde-1,3-phosphate (G1,3P), 3-phosphoglycerate (3PG), 2-phosphoglycerate (2PG), phosphoenolpyruvate (PEP), pyruvate (PYR), acetyl-CoA (AcCoA), acetyl phosphate (Ac-P), acetyl-AMP (Ac-AMP), citrate (CIT), glyoxylate (GOx), α-ketoglutarate (α-KG), succinyl-coenzyme A (SUC-CoA), succinate (SUC), fumarate (FUM), malate (MAL), oxaloacetate (OAA), 6-phosphogluconolactone (6PGNL), 6-phosphogluconate (6PGNT), ribulose-5-phosphate (RU5P), ribose-5-phosphate (R5P), xylulose-5-phosphate (X5P), seudoheptulose-7-phosphate (S7P), erythrose-4-phosphate (E4P), 2-keto-3-deoxy-6-phosphogluconate (KDPGNT), 3-deoxy-D-arabino-heptulosonate-7-phosphate (DAHP), 3-dehydroquinate (DHQ), 3-dehydroshikimate (DHS), shikimate (SHIK), chorismate (CHO), prephenate (PPA), phenylpyruvate (PPI), L-phenylalanine (Phe), L-tyrosine (Tyr), L-tryptophane (Trp).

Glucose is the preferred carbon source for *Escherichia coli *(*E. coli*); the presence of this monosaccharide inhibits the utilization of secondary carbon sources. This process is known as carbon catabolite repression, and the phosphoenolpyruvate: carbohydrate phosphotransferase system (PTS) is the main regulator of this response. The PTS components EI, HPr, EIIA^Glc^, EIICB^Glc ^encoded by the *ptsHIcrr *operon and the *ptsG *gene, as well as other important proteins like the adenylate cyclase (Cya) and the cAMP-CRP complex, are members of the carbon catabolite modulon regulating the activities of genes involved in carbon transport [1-5]. The inactivation of PTS has pleiotropic effects in *E. coli*. It has been demonstrated that the deletion of the *ptsHIcrr *operon drastically reduces the specific growth rate (μ) of the cell (from 0.7 to 0.1 hr^-1^) when growing on glucose as the only carbon source. In this type of strains, the overexpression of several genes involved in carbon utilization occurs as a response to carbon limited conditions. Accordingly, the cell is capable of coutilizing glucose with several other carbon sources that are not only carbohydrates. The absence of functional PTS (PTS^-^) permits the coexistence of glycolytic and gluconeogenic pathways in this type of strains [6-8]. PTS^- ^strains have been constructed with the goal of canalizing part of the phosphoenolpyruvate (PEP) not utilized in glucose transport to the synthesis of aromatic compounds [9-13]. However, as already mentioned, the absence of PTS causes poor growth on glucose. As such, PTS^- ^strains are not suitable for aromatic production purposes. Therefore, additional modifications are required to increase glucose transport and phosphorylation capacities in the PTS^- ^cell. Starting from the PTS^- ^PB11 strain (derived from JM101) in which the *ptsHIcrr *operon was deleted, several fast growing mutants on glucose have been selected using a chemostat fed with glucose at progressively higher rates [9]. One of these fast growing mutants (μ = 0.42 hr^-1^), named PB12 PTS^-^Glc^+^, uses the GalP and Glk proteins for glucose transport and phosphorylation, respectively, as has been previously demonstrated. Moreover, not only the carbon glycolytic flux in PB12 PTS^-^Glc^+ ^strain is highly increased as compared to PB11 [6], but also PB12 is capable of increasing the yield of aromatic compounds from glucose as compared to the wild type strain (Fig. 1) [13,14]. Finally, it has also been shown that strain PB12 maintains the capacity of coutilizing glucose and acetate with the consequent increase in its μ, similarly to what happens with the parental PB11 strain. These results indicate that in these PTS^- ^strains the carbon catabolite repression by glucose is not working for many carbon sources, which explains why strain PB11 is capable of coutilizing glucose and several other carbon sources [7,8]. In this report we analyze the capability of strain PB12 of simultaneously utilizing glucose and other carbon sources, as well as its capacity of enhancing the production of aromatic compounds due to this carbon coutilization capability.

## Results

### Coutilization of glucose and other glycolytic carbon sources by PB12

**Figure 2:**
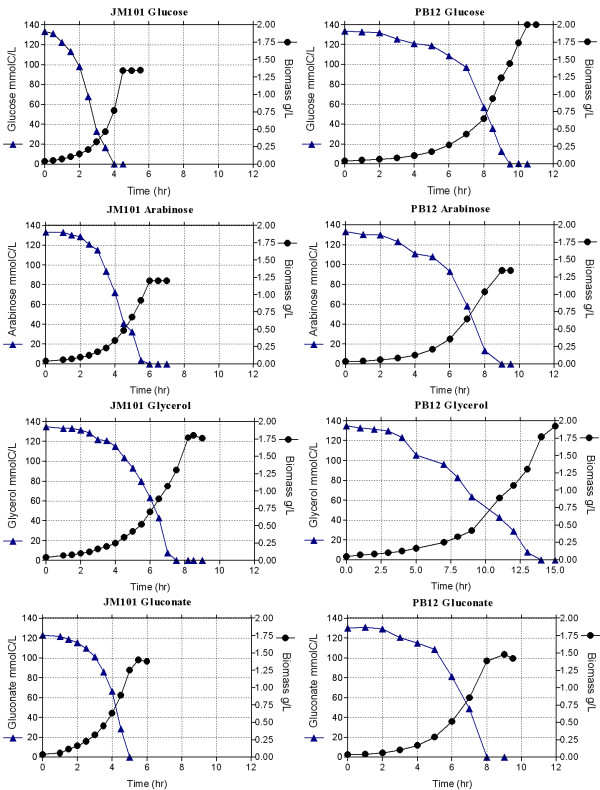
**Growth profile and substrate utilization of the wild type JM101 strain and its derivative PB12 on one carbon source**. Fermentor cultures of the two strains grown aerobically in M9 medium containing only one carbon source: glucose, gluconate, arabinose, or glycerol (4 g/L, equivalent to approximately 130 mmolC/L). Growth was monitored and the carbohydrates concentrations were assayed. The production of acetate was also measured. Mean values from two independent cultures are shown. Differences between values in these experiments were <10%.

**Figure 3:**
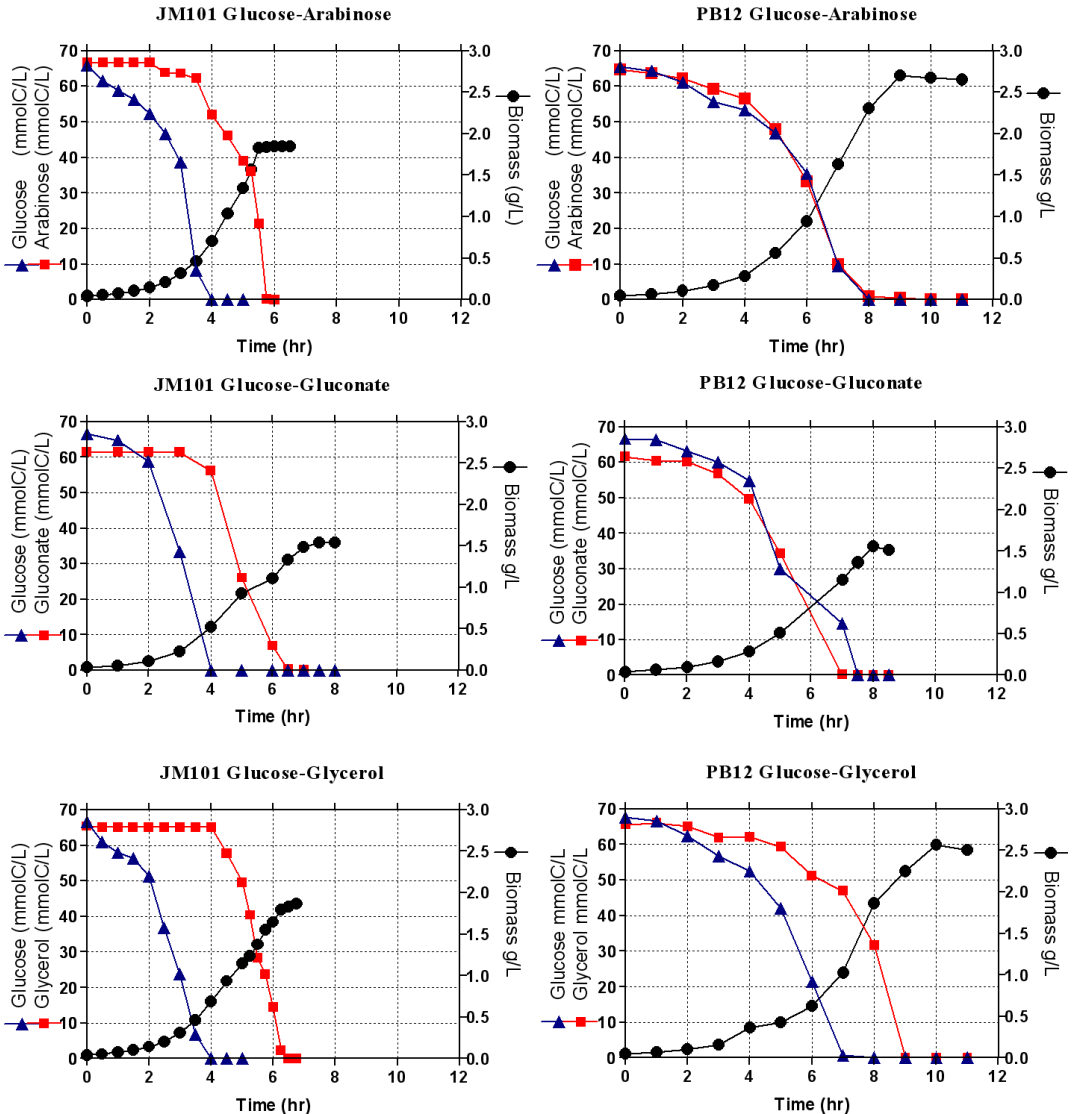
**Growth profile and substrate utilization of the wild type JM101 strain and its derivative PB12 on mixtures of two carbon sources**. Fermentor cultures of the two strains grown aerobically in M9 medium containing mixtures of glucose (2 g/L equivalent to 65 mmolC/L) and other carbohydrate (2 g/L, equivalent to approximately 65 mmolC/L). Growth was monitored and the concentrations of glucose and of the other carbohydrates were assayed. The production of acetate was also measured. Mean values from two independent cultures are shown. Differences between values in these experiments were <10%.

Strain PB12 presents a high glycolytic carbon flux when glucose is used as the only carbon source [6]. Therefore, the capacity of coutilizing several glycolytic metabolized carbon sources was analyzed in this strain. As shown in table 1 and figure 2, PB12 has the capacity of growing relatively fast on glucose and in other glycolytic carbon sources such as arabinose, glycerol and gluconate. When a mixture of glucose and one of these other carbohydrates was used in the fermentor as carbon sources, PB12 coutilized clearly in all cases, both carbohydrates three hours after the culture was started, while the parental strain JM101 exhibited different behaviors depending on the mixture present in the medium. Namely, glycerol uptake by JM101 initiated when glucose was completely exhausted, whereas for arabinose and gluconate, consumption of the second carbon source occurred when glucose was approximately 8.3 and 25 mmolC/L, respectively. Interestingly, only small amounts of acetic acid were detected when strain PB12 was grown on glycerol or in the glucose-glycerol mixture, whereas larger amounts were detected in the other mixtures (Tables 1 and 2). Carbon source utilization patterns and kinetic and stoichiometric parameters of the parental strain JM101 and its derivative PB12 grown in the different mixtures are shown in figure 3 and table 2, respectively.

**Table 1:** Kinetic and stoichiometric parameters for strain JM101 and its derivative PB12 PTS^-^Glc^+^, using one carbon source.

	**JM101**				**PB12**			
	
**Carbon sources**	**μ hr^-1^**	**Y_x/s _g/mmolC**	**qs mmolC/g_DCW _hr**	**acet_max _g/L**	**μ hr^-1^**	**Y_x/s _g/mmolC**	**qs mmolC/g_DCW _hr**	**acet_max _g/L**
Glucose	0.72	0.01800	71.88	0.44	0.42	0.0171	24.50	0.30
Arabinose	0.68	0.0066	155.84	0.50	0.50	0.0081	62.50	0.33
Glycerol	0.47	0.01070	43.90	0.18	0.32	0.0086	37.17	0.05
Gluconate	0.69	0.0049	80.00	0.55	0.51	0.0094	42.50	0.40

**Table 2:** Kinetic and stoichiometric parameters for strain JM101 and its derivative PB12 PTS^-^Glc^+^, using a mixture of two carbon sources.

	**JM101**				**PB12**			
	
**Carbon sources**	**μ hr**^-1^	**Y_x/s _g/mmolC**	**qs mmolC/g_DCW _hr**	**acet_max _g/L**	**μ hr**^-1^	**Y_x/s _g/mmolC**	**qs mmolC/g**_DCW_**hr**	**acet_max _g/L**
Glucose + Arabinose	0.72 (0.64)	0.0180 (0.0.012)	40.68 (7.64)	0.30	0.55	0.0144	38.19	0.30
Glucose + Glycerol	0.72 (0.45)	0.0171 (0.006)	43.11 (8.55)	0.13	0.50 (0.20)	0.0136 (0.017)	38.23 (9.24)	0.03
Glucose + Gluconate	0.72 (0.33)	0.00108 (0.0133)	70.33 (20.62)	0.49	0.51	0.0125	40.80	0.39

Different parameters (μ, qs and Yx/s, see Methods) were determined for strain JM101 and its derivative PB12 PTS^-^Glc^+ ^using mixture of two carbon sources. The numbers in parentheses indicate data for the specific strain in those stages in which only the remaining carbon source was available. In the case of JM101, the remaining carbon source was arabinose, glycerol, or gluconate, whereas glycerol was for PB12.

### Aromatic metabolite production in strain PB12 using different mixtures of carbohydrates as carbon sources

With the goals of increasing the production of the first aromatic intermediate metabolite of the shikimate pathway, 3-deoxy-D-*arabino*-heptulosonate 7-phosphate (DAHP) and of measuring the carbon flux in this pathway by avoiding its transformation into subsequent compounds, a mutation in the *aroB *gene in strain PB12 was constructed (details in Methods). This mutation cancels the transformation of DAHP into 3-dehydroquinate (DHQ) (Fig. 1). In addition, strain PB12 and its *aroB*^- ^derivative were cotransformed with the compatible plasmids pRW300*aroG*^*fbr *^and pCL*tktA*. These plasmids carry the *tktA *and *aroG*^*fbr *^genes that code for the transketolase A (TktA) and the AroG^fbr ^DAHP synthase insensitive to feedback inhibition by phenylalanine [9-14]. The transcription of *tktA *is under its constitutive promoter, while that of *aroG*^*fbr *^is under the control of the *lacUV *promoter that is induced with IPTG (details in Methods). TktA is involved in the synthesis of erythrose 4-P (E4P) one of the two precursors of DAHP. The second enzyme, AroG, is one of the three isoenzymes involved in the synthesis of DAHP from E4P and PEP (Fig. 1). It has been reported that overexpression of these two genes when present in multicopy plasmids enhances the production of aromatic compounds [9-16].

#### • DAHP production in PB12 *aroB*^-^(pRW300*aroG*^*fbr *^pCL*tktA*) resting cells

**Figure 4:**
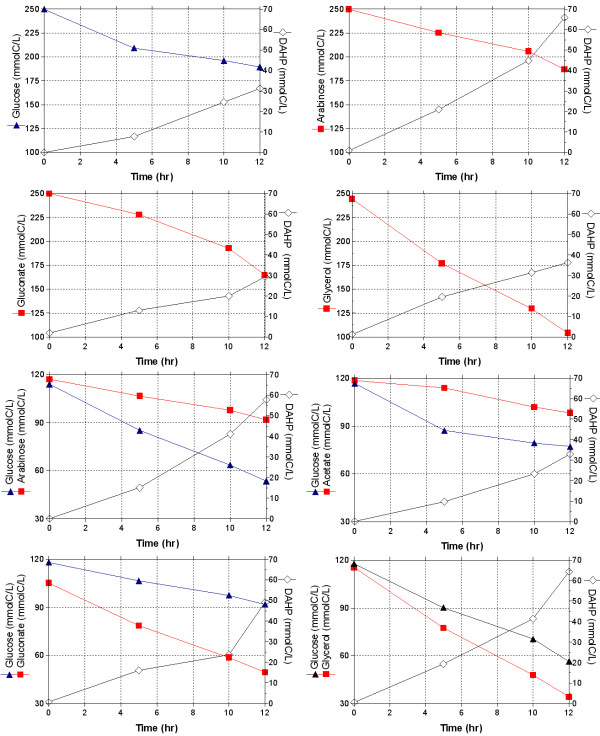
**Substrate utilization and DAHP production by resting cells of strain PB12 PTS^-^Glc^+^*aroB*^- ^(pRW300*aroG*^*fbr *^pCL*tktA*)**. Resting cells were incubated with the different carbohydrates in the conditions described in Methods. Carbon utilization and the production of DAHP were measured at different times after the addition of the carbohydrates. Mean values from two independent experiments are shown; differences between values in these experiments were <10%. The biomass value for the glycerol-glucose mixture was approximately 0.66 g/L, whereas for the rest of the carbon sources was 1.064 g/L (data not shown).

As can be seen in figure 4, DAHP production in strain PB12*aroB*^-^(pRW300*aroG*^*fbr *^pCL*tktA*) yielded similar values with glucose, gluconate and glycerol, whereas it doubled with arabinose. Interestingly, resting cell cultures on arabinose alone produced slightly more DAHP than with the glucose-arabinose mixture, and about the same amount of DAHP than that of the glucose-glycerol mixture. However, the productivity (qDAHP), in the glucose-glycerol mixture was superior to the one obtained for any other carbon source or analyzed combinations of carbon sources (Table 3). DAHP production and yields in strain JM101*aroB*^- ^carrying the same plasmids were 25 and 50 times lower, respectively, than the values obtained in strain PB12*aroB*^-^(pRW300*aroG*^*fbr *^pCL*tktA*) (data not shown).

**Table 3:** DAHP productivity and other important parameters determined for strain PB12 PTS^-^Glc^+^*aroB*^-^(pRW300*aroG*^*fbr *^pCL*tktA*) in resting cells.

**Carbon sources**	**q_DAHP _mmolC DAHP/g_DCW_hr**	**q_s _mmolC/g_DCW _hr**	**Y mmolC DAHP/mmolC carbon sources**
Glucose	2.44	4.72	0.52
Arabinose	5.09	4.93	1.03
Acetate	0.00	0.00	0.00
Gluconate	2.15	6.15	0.35
Glycerol	2.75	10.97	0.25
Glucose-Arabinose	4.52	6.72	0.67
Glucose-Acetate	2.57	4.69	0.55
Glucose-Glycerol	8.63	15.63	0.55
Glucose-Gluconate	3.80	6.44	0.59

#### • Aromatic intermediate production in the fermentor by the strain PB12 (pRW300*aroG*^*fbr *^pCL*tktA*)

**Figure 5:**
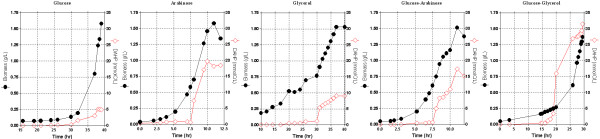
**DAHP production by strain PB12 PTS^-^Glc^+^(pRW300*aroG*^*fbr *^pCL*tktA*)**. Fermentor cultures of strain PB12 carrying these plasmids, grown aerobically in minimal medium, containing either a final concentration of 4 g/L equivalent to approximately 130 mmolC/L (in the case of one carbon source), or 2 g/L equivalent to approximately 65 mmolC/L, of each carbohydrate (when mixtures of two were utilized). Acetate concentrations were also measured in these fermentations and are reported in table 4. Mean values from two independent cultures are shown. Differences between values in these experiments were <10%. IPTG (0.1 mM) was included in the medium at the beginning of the fermentations.

**Figure 6:**
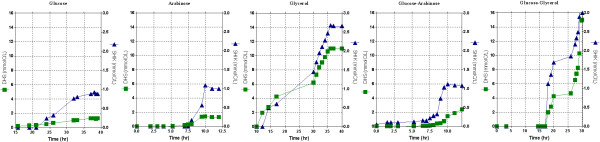
**DHS and SHIK production by strain PB12 PTS^-^Glc^+^(pRW30*aroG*^*fbr *^pCL*tktA*)**. The same fermentations for the production of DAHP were used for the measurements of DHS and SHIK intermediates. Mean values for two independent cultures are shown. Differences between values in these experiments were <10%.

Figures 5 and 6 show the production of DAHP, DHS and SHIK by strain PB12 (pRW300*aroG*^*fbr *^pCL*tktA*) when grown in the fermentor using different carbon sources. It is important to emphasize that the highest DAHP titer (31.33 mmolC/L, equivalent to 0.9 g/L) occurred in the case of the glucose-glycerol mixture, which almost doubled the amount obtained in arabinose and in the glucose-arabinose mixture and is three times higher that the amount produced in the glycerol culture (Fig. 5). Interestingly the highest production of DHS and SHIK also occurred in the glucose-glycerol culture and slightly lower in the glycerol glucose-glycerol mixture fermentation (Fig. 6). Table 4 shows the specific productivity values for these aromatic compounds that are also higher in the glucose-glycerol mixture. Other parameters determined during these fermentations, including acetate concentrations, are also presented in table 4.

**Table 4:** Kinetic and stoichiometric parameters determined for strain PB12 PTS^-^Glc^+ ^(pRW300*aroG*^*fbr *^pCL*tktA*) grown in the fermentor.

**Carbon Sources**	**μ hr**^-1^	**Y_X/S _g/mmolC**	**qs mmolC/g_DCW _hr**	**q_DAHP _mmolC_DAHP_/g_DCW _hr**	**q_DHS _mmolC_DHS_/g_DCW _hr**	**q_SHIK _mmolC_SHIK_/g_DCW _hr**	**acet_max _g/L**	**Yarom mmolC/mmolC**
Glucose	0.32	0.0093	34.33	1.3575	0.1464	0.1435	0.06	0.07
Arabinose	0.42	0.0066	64.61	3.5085	0.2359	0.0523	0.35	0.12
Glycerol	0.09	0.0081	11.15	0.6600	0.8000	0.2000	0.00	0.25
Glucose-Arabinose	0.42	0.0061	68.85	7.7967	0.3619	0.1367	0.30	0.13
Glucose-Glycerol	0.13	0.0076	17.11	9.0498	1.9112	0.9124	0.00	0.36

DAHP, DHS and SHIK productivities and other important kinetic and stoichiometric parameters determined in the fermentor using strain PB12 PTS^-^Glc^+^(pRW300*aroG*^*fbr *^pCL*tktA*). The expression of the *aroG*^*fbr *^gene was induced with IPTG.

## Discussion

The deletion of the *ptsHIcrr *operon exerts pleiotropic effects on the general physiology of the cell. PB11 grows very slowly (μ = 0.1 hr^-1^) when cultured on glucose as the only carbon source due to its inability to efficiently transport and phosphorylate glucose. Under such conditions, a nutrient scavenging stress response is induced which is responsible for the overexpression of many genes encoding carbon transport and metabolism proteins [7,8]. Due to the lack of the EIIA^Glc ^component, mainly responsible for catabolite repression, the PB11 strain is capable of coutilizing secondary carbon sources in the presence of glucose. The induction of several cAMP/CRP regulated genes in this strain suggests that the adenylate cyclase is still producing cAMP and unpublished evidence indicates that less cAMP is produced in the PB11 and PB12 strains as compared to the paternal JM101 strain (de Anda unpublished results). Nevertheless, there is apparently enough cAMP to allow the transcription of many cAMP-CRP regulated genes in these PTS^- ^strains [7,8]. Additionally, the transcription of the *rpoS *gene, that encodes the sigma factor RpoS for growth on non-optimal conditions, is upregulated in these strains. It is known that RpoS regulates various operons, including glycolytic genes, when *E. coli*. cells are grown in non-optimal conditions [8,17-22]. Strain PB12 PTS^-^Glc^+^, derived from strain PB11PTS^- ^in an adaptive evolution process, is capable of growing faster on glucose (μ = 0.42 hr^-1^). It has been previously demonstrated that when strain PB12 carries plasmids with the *tktA *and *aroG*^*fbr *^genes, the overexpression of these genes is responsible for canalizing part of the PEP, not utilized in glucose transport due to the absence of PTS, to the synthesis of aromatic compounds [9-14].

In this report we have demonstrated that strain PB12 is still capable of coutilizing other carbohydrates with glucose. This capacity was explored with the goal of further increasing the production of aromatic compounds in minimal medium, using strain PB12(pCL*tktA*, pRW300*aroG*^*fbr*^). The obtained results indicate that certain carbohydrate mixtures, especially glucose-glycerol, increase the capacity of aromatic metabolite production in both, resting cell and fermentor experiments. Such capacity was enhanced by more than six-fold (5.00 to 31.33 mmolC/L) in the case of DAHP in the fermentor experiments when compared to glucose as the only carbon source. Interestingly, less biomass (1.4 g/L) was produced in the glucose-glycerol mixture than in the other cultures and no acetate was detected in the glycerol and glucose-glycerol cultures. Furthermore, the yield of aromatics compounds obtained in this last mixture in batch conditions (0.36 mmolC/mmolC) is high and represents 53% of the maximum theoretical yield.

It is important to emphasize that the presence of these two carbohydrates as carbon sources increased the μ of PB12, as compared to the value obtained on glucose or glycerol. However, when the glucose-glycerol mixture was used by the PB12 strain carrying both plasmids – pCL*tktA *and pRW300*aroG*^*fbr *^– in the presence of IPTG where, as mentioned the best yield of aromatic compounds was obtained, then its μ was reduced as compared to the one obtained on glucose, although the lag phase was half of that obtained on glucose alone. It has been proposed that the presence of plasmids, which was confirmed during different fermentation steps, and the induction of the transcription of the *aroG*^*fbr *^gene with IPTG could cause a metabolic burden responsible for slower specific growth rates and longer lag phases, like the one obtained in the glucose culture [14]. The increased production of aromatic compounds is probably not only the result of a different carbon utilization capability but also of a modified carbon flux metabolism than the one present when the cell is grown only on glucose. Glycerol enters the glycolytic pathway as glycerol-3P and it is transformed to 3-dihydroxyacetone-phosphate (DHAP); this compound isomerizes to glyceraldehyde 3-phosphate (G3P) (Fig. 1). This metabolite is the substrate of the TktA enzyme that interconnects glycolysis with the non-oxidative branch of the pentose phosphate pathway and interconverts G3P and fructose-6P (F6P) into E4P and xylulose-5-phosphate (X5P) (Fig. 1) [22,23]. This situation could explain why glycerol and glucose, better than any other carbohydrate mixture, were capable of modifying and enhancing the carbon flux into E4P and possibly PEP, both precursors of DAHP, by increasing the production of aromatic intermediates including DHS and SHIK [24].

It is also interesting that no acetate was detected during the growth on glycerol and in the glucose-glycerol mixture in the fermentors, while acetic acid was produced in other mixtures, especially in those where the strains grew faster. This last result indicates that while PB12(pCL*tktA *pRW300*aroG*^*fbr*^) was coutilizing glycerol and glucose, it could also be utilizing previously produced acetate. Alternatively, the cells might not be producing large amounts of acetate in these conditions. This last alternative is in agreement with the low μ of this strain under the growth conditions in the fermentor. In accordance with this last proposition are the smaller qs values obtained for PB12(pCL*tktA *pRW300*aroG*^*fbr*^) strain on all the different carbon sources as compared to those obtained for strain JM101. In strain PB12(pCL*tktA *pRW300*aroG*^*fbr*^) such condition could diminish metabolic overflow which is responsible for acetate production. These results are interesting and indicate that the capacity of coutilizing certain carbohydrate mixtures is an important characteristic that allows this strain important carbon flux rearrangements. Namely in the case of the glucose-glycerol mixture, it diminishes its specific growth rate, increasing time of production, but enhancing the yields and productivities of aromatic compounds and reducing acetate production. These results indicate that strains that grow slower that the parental wild type could be better production strains because part of the carbon flux is utilized not for biomass and acetate production but for the synthesis of the desired metabolite. Further experiments, especially those conducted in enriched minimal media, usually utilized for the optimization of production processes [14-16,25], are required to demonstrate the real potential of this capacity under such growing conditions. Nevertheless, *E. coli *capability of coutilizing glucose with certain carbon sources in the absence of PTS is an important property that should be studied because it could have useful industrial possibilities.

Certainly, the use of *E. coli *strains lacking the PTS system, with additional mutations and in which several other genes coding for enzymes involved in the aromatic pathway are overexpressed, have been already described for the production of aromatic molecules like shikimate, a precursor of Tamiflu, an antiviral drug that could be used in a possible influenza pandemia. Optimized processes have been reported that allow the production of around 70 g/lt of shikimate with a yield of 0.26 g shikimate/g glucose and a total aromatic yield of 0.328 g/g glucose in feed-batch fermentations using enriched minimal medium [26]. Our group has already reported the utilization of strain PB12 derivatives to overproduce phenylalanine, using enriched minimal medium at the level of 1 lt. fermentors. In these conditions, the yield of L-phenylalanine from glucose increased 65% as compared to the isogenic PTS^+ ^strain [13,14]. Preliminary results using derivatives of strain PB12 in which the overexpresion of certain genes coding for enzymes involved in the shikimate pathway was achieved in enriched minimal medium at the level of 1 lt. fermentors, produce 7 g/lt of shikimate with a yield of 0.282 g shikimate/g glucose and a total aromatic yield of 0.39 g/g glucose (unpublished results). Additional mutations that we are incorporating in PB12 production derivative strains (like *pykA *and or *pykF *to modulate the glycolytic flux, and a mutation isolated in another PB12 derivative that allows very fast growth rates in acetate [μ = 0.9 hr^-1^]) could increase the production capabilities of PB12 and other PTS^- ^derivative strains. Finally, an additional consideration about the importance of characterizing these PTS^- ^strains is that in the scenario of a world influenza pandemia, it is important to develop national and regional capabilities to produce antiviral drugs like Tamiflu or Relenza and the next generations of antiviral aromatic molecules, using biotechnological strategies with optimized engineered strains.

## Methods

### Bacterial strains and plasmids

*E. coli *strains and plasmids used in this study are presented in table 5.

**Table 5:** *Escherichia coli *strains and plasmids used in this work.

**Strain or plasmid**	**Relevant genotype**	**Reference**
JM101	*supE, thi Δ(lac-proAB)*, F' *tra*D36 *proA*^+ ^*proB*^+ ^*lac*I^q ^*lac*ZΔM15	[31]
PB12	This strain was derived from PB11PTS^-^Glc^-^, a *ptsHIcrr *deletion derivative of strain JM101. PB12 has the same genotype of PB11 and at least three additional mutations (*arcB*, *rpoS *and a mutation responsible for the upregulation of genes involved in the ppGpp metabolism) that appeared during the selection of this fast growing mutant on glucose.	[7,9,21]
PB12*aroB*^-^	PB12*aroB::cat*	This work
pRW300	*aroG*^fbr ^is under the control of the IPTG inducible promoter *lacUV5*; carrying tetracycline resistance. Replication origin of pBR322.	[16] [10]
pCL*tktA*	*tktA *is under its constitutive promoter carrying spectinomycin resistance. Replication origin of pACYC184.	[16]

### Genetic procedures and recombinant DNA techniques

PCR reactions were performed using Platinum Taq polymerase according to the manufacturer recommendations (Invitrogen, Ca., USA). The DNA sequences of the oligonucleotides (DAB1 and DAB2) for the interruption of the *aroB *gene are presented in table 6. This table also includes the sequences of the oligonucleotides (SAB1 and SAB2) utilized for demonstrating the interruption of the *aroB *gene in strains JM101 and PB12. Mutagenesis of the *aroB *gene in strains JM101 and PB12 was obtained using the Datsenko and Warner methodology [27]. This method utilizes a λ phage derivative and low copy number plasmid pkD45. Details of the procedure have been described elsewhere [27].

**Table 6:** DNA sequences of the oligonucleotides utilized in this work.

**Name**	**Nucleotide sequence**
DAB1 (Forward)	5'-GAT GAT CAA AGC GCT AAA GTG GTT GCA AAC CAG ATT ATT CAC TGT GTA GGC TGG AGC TGC TTC G-3'
DAB2 (Reverse)	5'-GTC TTC TGG TTT GAA TTC ATC CAT TTA ACA CCC CAC TAA AAG CAT ATG AAT ATC CTC CTT AG-3'
SAB1 (Forward)	5'-GAT CTG CGG TTC GCC ACG TT-3'
SAB2 (Reverse)	5'-CAC CGC CGC GTG AAG TTC TGG-3'

### Growth conditions

#### • Batch cultures

M9 medium, consisting of (per liter): Na_2_HPO_4_, 6 g; KH_2_PO_4_, 3 g; NaCl, 0.5 g; NH_4_Cl, 1 g; MgSO_4_, 2 mM; CaCl_2_, 0.1 mM; Vit B1, 0.01 g, and glucose, 2 g, was utilized for growing fermentor inocula. A higher concentration of glucose or other carbohydrates (4 g/L, around 130.00 mmolC/L, depending of the molecular weight of the carbon source), was utilized in the fermentor (1 L, working volume: 0.75 L) studies when only one carbon source was employed. When two carbon sources were used, the same amount of each (2 g/L, approximately 65 mmolC/L) was employed. IPTG (0.1 mM) was added at the beginning of the fermentation. Tetracycline (30 μg/ml) and spectynomycin (100 μg/ml) were included in the medium for plasmid maintenance.

#### • Resting cells

ARO medium (per liter: K_2_HPO_4_, 14 g; KH_2_PO_4_, 16 g; NH_4_SO_4_, 5 g, and MgSO_4_, 1 g) with glucose (7 g/L) and 10 g/L yeast extract were utilized for growing the inocula in resting cell experiments. Inocula were washed twice with ARO medium and resuspended in 50 ml of the same medium in 250 ml shake flasks, lacking yeast extract and supplemented with aromatic aminoacids, vitamins, L-tyrosine (8 mg/L), L-tryptophan (4 mg/L), L-phenylalanine (8 mg/L), p-aminobenzoic acid (62 mg/L), dihydroxibenzoic acid (35 mg/L), and p-hydroxibenzoic acid (2 mg/L). Glucose or other carbohydrates were added at a final concentration of 7 g/L (around 228 mmolC). When a mixture of two carbohydrates was employed, equal amounts of both (3.5 g/L; approximately 114 mmolC) were utilized. For resting cell experiments, IPTG (0.1 mM) was added after one hour of fermentation. In these conditions *E. coli *cells do not grow but are still metabolically active and can canalize carbon skeletons into the production of aromatic compounds [12,28]. For plasmid maintenance, (30 μg/ml) and spectynomycin (100 μg/ml) were utilized.

### Kinetic and stoichiometric parameters

#### • Batch cultures

Data represent the average of at least three different cultures. Cell growth was measured by monitoring the optical density 600 nm (OD_600_) in a spectrophotometer (Beckman DU700). OD_600 _was converted into dry cellular weight (biomass concentration) using a standard curve (1 OD_600 _= 0.37 g/L of dry cellular weight). Specific growth rates (μ) were determined by fitting the biomass data versus time to exponential regressions. The biomass yield (Y_X/S_) was estimated as the coefficient of linear regression of biomass concentration versus substrate concentration (glucose, arabinose, glycerol, gluconate, or glucose plus other carbon source) in grams of biomass/mmolC of substrate(s). The specific carbon consumption rate (q_S_) was determined as the ratio of μ to Y_X/S_. Cells were grown in the fermentor on glucose alone or glucose and either arabinose, glycerol, or gluconate as carbon sources.

#### • Resting cells

Specific glucose consumption and product formation rates were determined for each strain by linear regression of four data points over the 12-hr time span of an experiment. Constant DAHP production and carbohydrate consumption rates were found for all strains indicating that carbon catabolism was in a physiological quasi-steady-state [12,28,29]. For *aroG*^*fbr*^overexpression, cells were grown in the presence of 0.1 mM IPTG.

### Analytical methods

Metabolite concentrations were determined with an HPLC system (600E quaternary bomb, 717 automatic injector, 2410 refraction index, and 996 photodiode array detectors, Waters, Milford, MA). For determination of D-glucose, L-arabinose, D-glycerol, 3-dehydroshikimate (DHS), shikimate (SHIK) and acetic acid, an Aminex HPX-87H column (300 7.8 mm; 9 Am) (Bio-Rad, Hercules, CA) was used. Running conditions were: mobile phase, 5 mM H_2_SO_4_; flow, 0.5 mL/min, and temperature, 50°C. Under these conditions glucose and acetic acid were detected by refraction index. D-gluconic acid was determined by enzymatic assay (Boehringer-Mahheim, Darmstadt, Germany). DAHP concentrations were determined using the thiobarbituric assay [30].

## Conclusion

PTS^- ^strains are capable of coutilizing different mixtures of carbon sources due to the lack of glucose catabolite repression. The PTS^- ^strain PB12, carrying multicopy plasmids with *tktA *and *aroG*^*fbr *^genes, was capable of coutilizing glucose and glycerol. This capacity diminished its specific growth rate but increased six-fold the production of DAHP, and the production of acetate was not detected. These results suggest that at least in this PTS^- ^strains in these growing conditions, slower growth rates allow better carbon utilization strategies for production purposes, diminishing metabolic overflow.

## Competing interests

The author(s) declare that they have no competing interests.

## Authors' contributions

KM and FB designed experiments, analyzed the results and wrote the manuscript. KM carried out the experiments while GH and RA helped with analytical and fermentation experiments. AE, GG and OTR critically revised the results and the manuscript. All the authors have read and approved the publication of the manuscript.
